# Efficiency and Temporal Reasoning in Transformer-Based Video Object Detection: A Systematic Review

**DOI:** 10.3390/jimaging12090419

**Published:** 2026-09-05

**Authors:** Yiannis Keravnos, Anastasia Ioannou, Andreas Papadopoulos, Vicky Papadopoulou Lesta

**Affiliations:** 1 Cyprus Research and Innovation Center Ltd. (CYRIC), 2643 Nicosia, Cyprus; a.papadopoulos@cyric.eu; 2 Department of Computer Science and Engineering, European University Cyprus, 2404 Nicosia, Cyprus; an.ioannou@euc.ac.cy (A.I.); v.papadopoulou@euc.ac.cy (V.P.L.)

**Keywords:** video object detection, vision transformers, PRISMA, systematic literature review, lightweight architectures, temporal modeling

## Abstract

This PRISMA 2020 systematic literature review analyzes efficient transformer-based video object detection (VOD). Across five databases through August 2025, 1343 records were identified, with 16 studies meeting the final inclusion criteria after full-text screening. Backward citation tracking added five additional eligible studies, yielding a small final evidence base of 21 studies (22 reports). This review’s major contributions are (i) a focused narrative synthesis at the intersection of efficient model design, temporal modeling, and video object detection transformers, due to methodological heterogeneity in the corpus; (ii) ROB-CVA, a proposed risk-of-bias framework for computer vision; and (iii) an evidence-based analysis of temporal modeling strategies and evaluation practices. Furthermore, we examine evaluation inconsistencies, including dataset fragmentation and risk-of-bias. Fifty-two percent of studies were rated as low risk-of-bias using the review-specific, non-validated ROB-CVA framework, driven mainly by non-public code and datasets; this distribution is sensitive to our risk-of-bias aggregation rule. The certainty of evidence is low to moderate. The review outlines open challenges and future directions. This review is registered on OSF (doi: 10.17605/OSF.IO/Q9PJ4), supported by project AEOLUS (PHD IN INDUSTRY/1123/0145), and funded by the Republic of Cyprus through the Research and Innovation Foundation.

## 1. Introduction

Video object detection (VOD) is the task of localizing and classifying objects in video sequences and is fundamental to computer vision (CV) applications such as autonomous driving, surveillance, robotics, and smart city infrastructure [1]. The proliferation of video data from mobile devices, surveillance systems, and edge sensors, alongside an increased dependency on cloud resources, has intensified the demand for scalable, efficient models capable of real-time inference.

### 1.1. Architectural Evolution of VOD

Conventional deep neural networks, while highly accurate, are typically designed for high-performance GPUs and often fail to meet the strict latency, memory, and power requirements of embedded platforms [1,2,3]. Applying image-based detectors directly to video data not only introduces inefficiencies, leading to redundant computation, but also compromises performance in challenging scenarios such as motion blur and occlusion. The lack of temporal correlation exploitation between frames further exacerbates this trade-off. While traditional VOD frameworks address such issues through explicit flow-based alignment (FGFA) [4] or complex global–local memory aggregation (MEGA) [5], these methods often rely on computationally expensive modules that are incompatible with the strict latency requirements of edge hardware. Consequently, there is a critical need for architectures capable of temporal reasoning through more efficient means.

Transformer-based architectures have emerged as a promising direction for VOD due to their ability to model long-range dependencies and capture temporal dynamics more effectively than CNN-based approaches. Their attention mechanisms offer interpretable relational patterns and enable rich long-range reasoning, making them well suited for video-based tasks. However, pure transformer architectures remain computationally and memory intensive, raising questions about their feasibility for edge deployment.

Recent detector families such as DETR [6] and subsequent real-time variants (RT-DETR v1–3) [7,8,9] illustrate the shift towards transformer-based detectors while simultaneously motivating research into lightweight and hybrid designs, compact backbones, and efficient attention mechanisms.

To delineate the scope of this review, we explicitly focus on architectures with temporal modeling capabilities. Stateless (frame-by-frame) approaches are treated as beyond this scope, not due to a lack of merit but as a decision to maintain coherence in analyzing architectures that explicitly exploit temporal dependencies.

### 1.2. Temporal Modeling and Quantifying Efficiency

Despite the centrality of “lightweight” claims to describe efficiency in this systematic literature review (SLR), relevant terms remain inconsistently defined across the literature. Authors variously apply it to reduced parameter count, improved inference speed, reduced floating-point operation (FLOP) counts, or lower energy consumption. This ambiguity prevents independent verification of efficiency claims and complicates cross-study comparison, a problem well recognized in the broader efficient deep learning literature yet unresolved at the community level. Its implications for cross-study comparability are analyzed in Section 5.2.1, and concrete proposals for standardized thresholds are offered in Section 5.4 as part of this review’s recommendations for future reporting practice. Included studies are characterized according to the efficiency metrics their authors measure and report relative to a stated baseline, consistent with the inclusion criterion applied during screening.

For the purposes of this review, explicit temporal modeling refers to any architectural mechanism designed to aggregate, propagate, or reason over information across more than one frame during inference. This includes, but is not limited to, feature memory banks, recurrent states, temporal attention modules, graph-based spatiotemporal aggregation, and multi-frame fusion modules. Models that apply a detection architecture independently to each frame without any cross-frame information exchange are classified as frame-by-frame inference and do not satisfy this criterion, regardless of whether they are evaluated on video data. This distinction is motivated by the review’s focus on architectures that explicitly exploit the temporal structure of video, rather than those that treat video as an unordered collection of static images.

This review further distinguishes two classes of mechanism within temporal modeling. Temporal aggregation refers to mechanisms that combine features or predictions from multiple frames into a single representation, such as memory banks and multi-frame fusion modules. Temporal reasoning refers to mechanisms that infer relationships or dependencies between frames, such as graph-based spatiotemporal reasoning and cross-frame attention. These two classes are not mutually exclusive as a given mechanism may combine both.

### 1.3. Identified Gap

Several surveys have addressed VOD and related topics [10,11], lightweight detection [12,13,14], transformer-based small object detection [15], or long-term historical trends [16]. Yet, these works treat lightweight design, temporal modeling, and transformer architectures as separate research threads. To date, to the best of our knowledge and based on our examination of the cited surveys and reviews, we have not identified any study that synthesizes the complete intersection of transformer architectures, VOD, temporal modeling, and efficiency. Table 1 summarizes the identified gap by showing existing surveys and how they treat these topics in isolation.

The increasing diversity of temporal modeling and efficiency reporting practices in recent VOD research motivates the need for a structured, reproducible synthesis. Accordingly, this review adopts the Preferred Reporting Items for Systematic Reviews and Meta-Analyses (PRISMA) 2020 [17] guidelines to ensure transparency, reproducibility, and methodological rigor. Moreover, it proposes a risk-of-bias assessment (ROB-CVA) adapted from Prediction model Risk Of Bias ASsessment Tool for AI (PROBAST-AI [18]) for the assessment of risk of bias in the selected studies.

**Table 1:** Gap-justification table summarizing existing surveys. Column headers represent: 1: Video, 2: Efficiency, 3: Transformers, 4: Temporal Modeling.

Study (Year)	1	2	3	4	Primary Focus
Ren et al. (2018) [19]		✓			Edge computing
Zhao et al. (2019) [20]					General deep learning
Bouguettaya et al. (2019) [12]		✓			Lightweight CNN
Jiao et al. (2022) [10]	✓				Video object detection
Zheng et al. (2022) [11]	✓				Video object detection
Chen (2023) [21]	✓	✓		✓	Temporal redundancy
Cheng et al. (2023) [22]	✓	✓			Small object detection
Li et al. (2023) [23]	✓	✓			Knowledge distillation
Miri Rekavandi et al. (2026) [15]			✓		Transformers (small objects)
Ghahremannezhad et al. (2023) [24]	✓				Traffic videos
Zou et al. (2023) [16]					Historical survey (20 years)
Mittal (2024) [13]		✓			Edge devices
Miri Rekavandi et al. (2025) [25]					Video small objects
El Zeinaty et al. (2025) [14]		✓			TinyML
This Review (2026)	✓	✓	✓	✓	VOD + Efficiency + Transformers + Temporal modeling

## 2. Motivation

This review is motivated by the absence of a synthesis connecting efficiency, temporal modeling, and transformer-based VOD, as established in Section 1. Without such a synthesis, design trade-offs across these dimensions remain scattered across sub-domain-specific literature, complicating comparison across studies.

Key advances in lightweight design, temporal attention, and real-time deployment have occurred predominantly after 2020, following the introduction of end-to-end transformer detectors [6]. This systematic review focuses on the 2020–2025 period to capture the emergence and rapid maturation of transformer-based VOD. Studying this specific period enables a coherent synthesis of the field from its most formative stage to its current state, focusing on the intersection of efficiency, temporal modeling, and transformer-based detection.

### Objectives

The objective of this study is to consolidate and critically evaluate the current landscape of lightweight VOD, with a particular emphasis on transformer-based architectures. Given the increasing demand for efficient, real-time video analytics in resource-constrained environments, this review aims to identify key design principles and challenges, as well as optimization strategies associated with lightweight VOD. By synthesizing existing research, the review seeks to uncover performance trade-offs, highlight gaps in the literature, and propose directions for future work.

To achieve this, the review is guided by the following research questions (RQs):

RQ1 What architectural designs and lightweight techniques are currently used in transformer-based video object detection models?
RQ2 How do these models exploit temporal information while maintaining computational efficiency?
RQ3 What optimization strategies are most effective for reducing computational complexity?
RQ4 How do these models perform across standard datasets, and what trade-offs exist between accuracy, speed, and model size?

## 3. Methodology

This systematic review was conducted and is reported in accordance with PRISMA 2020 [17]. The completed PRISMA 2020 checklist is provided as Supplementary Materials.

### 3.1. Eligibility Criteria

The eligibility criteria were defined in accordance with the research questions and PRISMA guidelines and are presented in Table 2.

### 3.2. Information Sources

The following electronic databases were systematically searched: IEEE Xplore Digital Library (searched 6 August 2025), ACM Digital Library (searched 2 August 2025), ScienceDirect (Elsevier) (searched 20 August 2025), SpringerLink (searched 25 August 2025), and Scopus (searched 14 August 2025). A final consistency check across all five completed searches was performed on 28 August 2025. To maintain methodological rigor and ensure replicability, the formal search was restricted to peer-reviewed databases.

### 3.3. Search Strategy

A unified base query was developed and adapted to database-specific syntax and technical constraints. For instance, queries were simplified for ScienceDirect to respect its 8-connector Boolean limit, while Springer Link and ACM were manually verified or filtered by discipline (e.g., Computer Science) for noise mitigation caused by full-text indexing and inconsistent categorization. To ensure the relevance and quality of retrievals while also speeding up the screening process, database-specific filters (publication year, document type, and discipline where available) were applied. A uniform date range of 2020–2025 was enforced, with document types restricted to English peer-reviewed articles and conference papers.

The Architecture criterion applies a complete transformer encoder or decoder stack definition from Vaswani et al. [26]. This definition is chosen because it can be consistently applied across studies to answer RQ1. Isolated attention mechanisms, non-local blocks, relation networks, and set-based prediction heads alone do not meet this definition. Hence, studies that lack the defined criterion are excluded. In doing so, hybrid architectures like many seminal studies (e.g., DETR [6]) remain eligible, while the impact on the search completeness was deemed minimal.

#### 3.3.1. Search Terms and Query Construction

Search terms were developed iteratively through preliminary searches and consultation of related SLRs, during which retrieved records were inspected to identify missing synonyms and adjust Boolean logic. The query construction combined keywords, synonyms, Boolean operators, and wildcards to maximize the sensitivity of the search. Subsequent iterations helped balance the precision and recall of search results to maintain relevance. Various synonyms or related terms were piloted but excluded when they introduced excessive noise or redundancy based on the research questions and scope of the review. Table 3 summarizes the retained and excluded terms.

#### 3.3.2. Boolean Operators, Truncation, and Wildcards

Boolean operators (AND, OR) were used to combine search terms effectively. Related terms were linked with OR to broaden the search, while distinct concepts were linked with AND. Truncation (*) was applied to root words to capture variations (e.g., “efficien*” to find “efficient”, “efficiency”). Wildcards (?) were employed for spelling variations (e.g., “real?time”) and double quotation marks for exact phrase matching. Although support for wildcards, truncation, and Boolean operators varied across databases (e.g., ScienceDirect’s limit on connectors and lack of wildcard support), the core query was adapted per platform while preserving the same conceptual components.

#### 3.3.3. Search Query

Table 3 combines thematic sub-strings derived from the research questions. A unified query approach ensured the core search string was used in all databases with minor adjustments for transparency and reproducibility (Listing 1).

**Listing 1.** Core Search String(lightweight OR efficient OR “real?time” OR edge) AND (video OR stream* OR sequence OR temporal) AND (“object detection” OR “object recognition”) AND (transformer* OR ViT)

### 3.4. Selection Process

**Figure 1:**
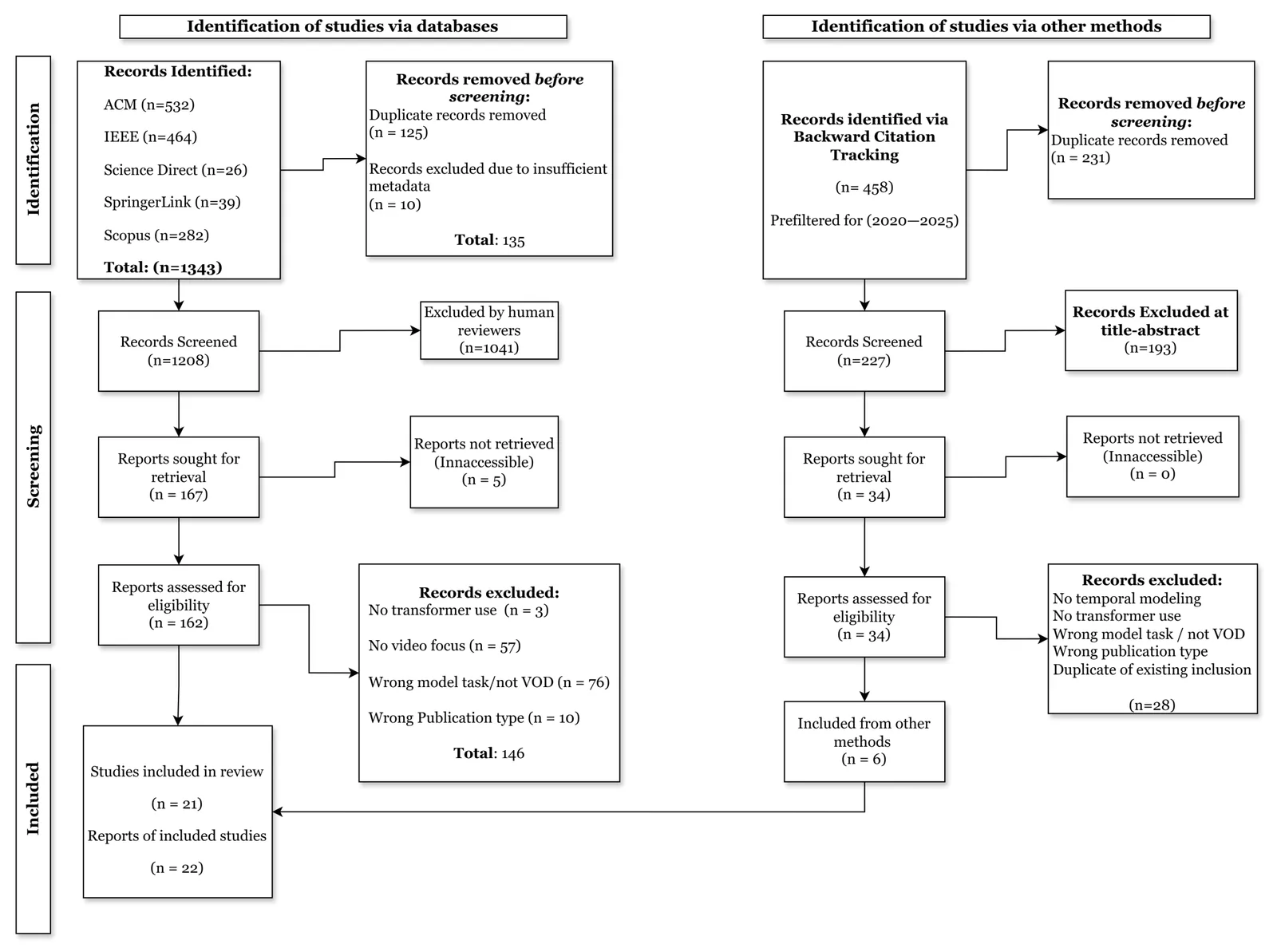
PRISMA Flow Diagram for summarizing the review process. The left column shows the main review process, while the side on the right summarizes the backward citation tracking process.

All records retrieved from the selected databases were exported into Zotero [27], where duplicates were manually removed. We conducted all stages of screening using Rayyan [28] independently with Blind Mode enabled. Blind Mode ensures reviewers had no indication of each other’s decisions. Conflicts were resolved through discussion or consultation when necessary to reach a consensus. Based on the PRISMA 2020 guidelines, a flow diagram (see Figure 1) is constructed to summarize the number of records identified, screened, excluded, and included, with documented reasons at each stage, while the screening stages are also listed below.

Screening stages:

- Title and abstract screening: All non-duplicate records were screened against the predefined eligibility criteria.
- Full-text review: Full texts of potentially eligible studies were retrieved and assessed independently; reasons for exclusion were recorded.
- Final inclusion: Only studies meeting all of the eligibility criteria were retained for data extraction. A checklist was created to aid this stage (see Appendix A, Table A1).

### 3.5. Data Extraction

A standardized data extraction form was developed in advance and piloted on a subset of included studies to ensure completeness and clarity. Data were extracted independently by two reviewers, with discrepancies resolved through discussion and consensus. No additional automation tools were used, and no study authors were contacted for additional information.

### 3.6. Data Items

The data extraction form is designed to select the following information:

- Bibliographic Information: Author(s), publication year, venue, DOI.
- Architecture Category: Model family or architectural bin, as defined in Section 4.
- Temporal Modeling Strategy: Type of explicit temporal mechanism employed, consistent with the eligibility criterion in Table 2.
- Datasets: All datasets used for primary training/evaluation, including specific subsets or versions, excluding ablation study evaluations.
- Efficiency and Complexity Metrics: frames per second (FPS) or latency, floating point operations (FLOPs), and parameter count where available.
- Accuracy Metrics: mean average precision (mAP) values at reported IoU thresholds, or sizes.
- Hardware/Software Environment: GPU specifications.
- Code Availability: Public availability of source code or pretrained models.

For each study, all reported values corresponding to the defined outcome domains were extracted when available. When information was missing or unclear, values were recorded as “not reported”. Values inferred from dataset convention or model family defaults, or approximated from a source study’s figures rather than its reported text, were treated as not reported in the main synthesis (see Section 4.4 and Section 4.5) and recorded as “NR”. These derived values are retained separately in Table A3, labeled as reviewer-derived and excluded from the primary synthesis. A discussion of this exclusion and its implications is provided in Section 5.3.4. No other assumptions or imputations were made. Capturing hardware/software environment details was considered essential, as efficiency claims (e.g., FPS) depend strongly on computational resources.

### 3.7. Risk of Bias Assessment

The examined scope of this review can be affected by several sources of bias. Based on concerns commonly discussed in machine learning and computer vision methodology, the following bias categories were identified a priori:

- Hardware-Dependent Performance Bias: Efficiency metrics such as FPS depend heavily on hardware, yet reporting is inconsistent, limiting comparability and transferability.
- Dataset Selection Bias: Many studies rely on limited or highly curated datasets that do not reflect real-world VOD challenges.
- Metric Reporting Bias: Efficiency metrics are reported inconsistently across studies, with varying indicators and hardware contexts, limiting direct cross-study comparability.
- Baseline Comparison Bias: Comparisons are frequently conducted under non-identical conditions (e.g., varying resolutions, batch sizes, or hardware), which may favor the proposed method.
- Reproducibility Bias: Missing details on training and inference configurations, random seeds, or code availability hinder reproducibility.

To systematically evaluate these risks, we adapted PROBAST-AI [18], a quality, risk of bias (ROB), and applicability assessment tool originally for biomedical applications with AI, to be used in computer vision research. Although originally developed for clinical AI, its structured domains, datasets, inputs, outcomes, and analysis can be transferred well to the methodological challenges of VOD. Our adaptation, named ROB-CVA (risk of bias for computer vision assessments), emphasizes (i) hardware transparency and reproducibility, (ii) fair baseline comparisons, and (iii) consistent and clearly defined metric reporting. The adapted criteria are shown in Table 4.

ROB-CVA adapts the four-domain structure of PROBAST + AI. The participants and data sources domain is retained as Datasets, with population representativeness reframed as dataset public availability and diversity. The predictors domain is retained as Inputs, covering resolution, preprocessing, augmentation, and modality-specific parameters. The outcome domain is retained as Evaluation Metrics, covering consistent definition and application of accuracy metrics. The analysis domain is split into Methodology and Reproducibility and Fair Comparison and Generalizability, separating architecture and reproducibility reporting from baseline comparison validity, as these usually represent distinct sources of bias in CV evaluations. Notably, Applicability is not rated as a separate dimension in ROB-CVA. It is instead incorporated as a single criterion within Fair Comparison and Generalizability. The model development/evaluation distinction used in PROBAST + AI is not retained, as CV studies to which ROB-CVA applies typically report both the development and evaluation of the proposed model within the same publication, rather than as separate development and external validation studies.

ROB-CVA has not undergone external validation. Its domain structure and aggregation rule were developed for this review and have not been tested for inter-rater reliability, sensitivity to alternative aggregation schemes, or applicability beyond the corpus assessed here.

As with other sources of disputes, the two reviewers conducted the risk of bias assessment manually and independently, without the use of any automation tools and resolving disagreements through discussion. Studies were classified as having Low, Moderate, or High risk of bias. Overall risk-of-bias ratings were determined using a conservative rule-based aggregation, i.e., Overall = High if at least one domain was rated High; Overall = Moderate if at least one domain was Moderate and none High; and Low risk only when all domains were rated Low.

### 3.8. Effect Measures

No formal effect measure was computed, consistent with the narrative synthesis approach adopted (see Section 3.9). Reported outcomes (e.g., mAP, FPS/latency, FLOPs, parameter count) are presented per study rather than pooled across studies.

### 3.9. Data Synthesis Approach

Data from included studies were synthesized using a structured narrative approach supported by quantitative tabulation. Studies were grouped according to architectural category, temporal modeling strategy, and evaluation protocols to ensure that comparisons were made within conceptually coherent groups.

Quantitative results (mAP, FPS/latency, FLOPs, and parameter counts) were summarized in tables to enable direct cross-study comparison and to highlight accuracy/efficiency trade-offs. Missing or inconsistently reported values were indicated with a dash (“–”) in the presented tables, and no data transformations or imputations were performed. Superscript annotations provide additional context where applicable.

The narrative synthesis examined similarities and differences in study design, datasets, evaluation protocols, and reported outcomes, with particular attention to how architectural choices and temporal modeling strategies influenced performance and efficiency. Heterogeneity in datasets, hardware, and reporting practices precluded meta-analysis; therefore, narrative synthesis with quantitative summaries was the most appropriate and transparent method for this domain. PRISMA 2020 acknowledges that SLRs may use narrative summaries and requires that the results of each synthesis, meta-analysis or otherwise, include concise summaries of study characteristics and risk of bias to support interpretation (Item 20a; [29]).

### 3.10. Reporting Bias Assessment

Reporting bias was assessed narratively, examining dataset and outcome availability, code and configuration disclosure, and consistency of efficiency metric reporting across the corpus (Section 4.6). Statistical methods require pooled effect sizes, which were not computed for the reasons given in Section 3.9.

### 3.11. Certainty Assessment

A formalized framework such as GRADE was not applied. Given the heterogeneity described in Section 3.9 and the narrative nature of this synthesis, a conventional GRADE assessment was unsuitable for our objectives. Instead, certainty in the body of evidence was judged narratively based on the consistency of evaluation protocols, the extent of cross-study benchmark overlap, and the reproducibility limitations identified in Section 4.7.

### 3.12. Backward Citation Tracking

To mitigate the risk of omitting relevant studies absent from the primary database search due to non-standard terminology or indexing limitations, backward citation tracking (BCT) was conducted as a supplementary retrieval step following completion of primary screening. BCT is recognized in PRISMA 2020 as a recommended method for identifying additional eligible records through the reference lists of included studies.

The reference lists of all studies included after primary screening were exported from Connected Papers as a consolidated CSV file and moved to Zotero for duplication removal and candidate identification. The resulting records were screened against the same eligibility criteria defined in Table 2, the two independent reviewers as outlined in Section 3.4, with conflicts resolved through discussion. Title and abstract screening was applied to all candidate records, followed by full-text review of studies appearing to meet the inclusion criteria. Studies confirmed eligible were subjected to the same data extraction and risk-of-bias assessment procedures described in Section 3.5 and Section 3.7 and are incorporated into the final corpus alongside the primary-search studies.

## 4. Results

### 4.1. Study Selection

Records were retrieved from IEEE Xplore (*n* = 464), ACM (*n* = 532), ScienceDirect (*n* = 26), SpringerLink (*n* = 39), and Scopus (*n* = 282), yielding 1343 records published between January 2020 and August 2025. Following removal of duplicates (*n* = 125) and records excluded due to insufficient metadata (*n* = 10), 1208 records were screened by title and abstract. Of these, 167 full-text studies were sought for retrieval, of which five were inaccessible, leaving 162 records for eligibility assessment. From full-text records, 146 were excluded for reasons that include no transformer use, lack of video focus, wrong model task, and wrong publication type. The final main corpus comprises 16 included studies, each corresponding to a single published report. The full selection process is summarized in Figure 1. The inclusion/exclusion checklist applied at the full-text stage is provided in Appendix A (see Table A1).

The largest exclusion category was “wrong model task or not VOD focus”. Where studies could fit multiple exclusion criteria, the most salient reason was recorded to maintain disjoint categories.

The high exclusion rate (98.8%) reflects the specificity of the eligibility criteria. The 16 included studies from the primary corpus span five architectural families and five application domains, while the complete corpus of included studies is expanded following backwards citation tracking (see Section 4.1.2).

#### 4.1.1. Eligible Exclusions

Upon full-text review, 20 studies were identified that initially appeared to meet the inclusion criteria but were ultimately excluded (see Appendix B, Table A2). These studies were excluded either because they performed frame-by-frame inference without any cross-frame aggregation, propagation, or memory mechanism, failing the temporal modeling criterion, or because the study did not clearly establish or reference a video modality, failing the task criterion despite employing a transformer-based detection architecture. Both exclusion reasons stem from a failure to unambiguously address RQ2, which concerns the exploitation of temporal information in VOD, and were applied to maintain analytical consistency in the corpus.

#### 4.1.2. Backward Citation Tracking Results

Backward citation tracking (BCT) of the reference lists of the 16 primary-corpus studies identified 458 candidate records after applying the 2020–2025 date filter. Following de-duplication in Zotero, 227 unique records were screened by title and abstract, of which 34 were advanced to full-text review. Of these, six met all eligibility criteria, while two of those records appear to correspond to a conference and journal version of the same study. The two records were treated as a single study, with data extracted from the more complete report. BCT therefore yielded five additional included studies (six reports), and the final corpus comprises 21 included studies (22 reports). The PRISMA flow diagram reflecting the BCT branch is presented in Figure 1.

### 4.2. Study Characteristics

**Figure 2:**
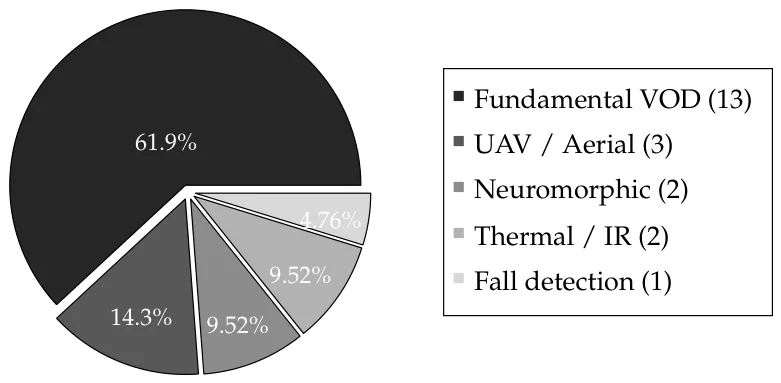
Distribution of included studies by application domain (*n* = 21).

Key characteristics of the included studies are summarized in Table 5. These include bibliographic information such as application domain and publication venue and year. The distribution of application domains is visualized in Figure 2.

#### Corpus-Reported Evaluation Datasets

While early developments in lightweight VOD were driven by standardized, general-purpose benchmarks, recent trends show a marked shift toward domain-specific evaluations. This SLR examines this transition in respect to benchmarking datasets through a detailed analysis of the current evaluation landscape.

Based on the examined corpus, video object detection benchmarks serve a dual role: they provide standardized evaluation foundations and implicitly encode assumptions about deployment contexts, such as object scale, motion profiles, and annotation density. ImageNet-VID [51,52] remains the canonical benchmark for general-purpose VOD despite its sparse annotation density. Notably, it is observably supplemented or replaced in the literature by domain-specific datasets for this reason, with higher annotation density alternatives. Namely, the following well-documented datasets in broader CV literature sparsely appear in the selected studies as well:

- VisDrone-VID [53] focuses on UAV-captured footage, where scale variation and aerial distortion pose significant challenges for detecting densely packed, small objects, representing a substantially harder evaluation surface than ImageNet-VID due to scale variation and aerial perspective distortion.
- UA-DETRAC [54] emphasizes complex, real-world traffic scenarios characterized by high-density vehicular flow, frequent occlusions, and varying environmental lighting, offering a more constrained yet realistic surveillance-oriented evaluation surface.
- KITTI [55,56] targets autonomous driving scenarios with stereo and LiDAR-aligned video, annotated for vehicles, cyclists, and pedestrians, and is distinctive in pairing visual detection with depth and motion cues for computer vision tasks beyond simply VOD.
- Gen1 [57]/1MPx [58] datasets serve as specialized benchmarks for event-based detection, focusing on high-frequency, low-latency streams that prioritize motion priors and temporal continuity over traditional frame-based semantic density.
- FL-Drones [59] refined by Dogfight [60] addresses specific challenges in detecting flying objects in diverse environments, providing a targeted evaluation surface for drone-to-drone or drone-to-ground visibility.
- NPS-Drones [61] provides a specialized benchmark for multi-target tracking and detection in aerial scenarios, emphasizing the challenges of small-object tracking in dynamic UAV flight paths.
- SIRSTD (Single-frame Infrared Small Target Detection) suite of datasets addresses the challenge of identifying dim targets in low-contrast, complex backgrounds, where detection reliability depends on thermal signatures rather than standard visual features.
- CAUCAFall [62] and DiverseFALL10500 [63] provide controlled evaluation surfaces for human fall recognition.

While broader VOD surveys also document YouTube-BoundingBoxes [64] as a large-scale general-purpose benchmark, it does not appear as an evaluation surface in the transformer-based lightweight VOD literature covered by this review.

### 4.3. Risk of Bias

All included studies were evaluated using the proposed ROB-CVA framework described in Table 4 covering five domains: Datasets, Inputs, Evaluation Metrics, Methodology and Reproducibility, and Fair Comparison and Generalizability. Table 6 presents a traffic lights plot to illustrate the ROB assessment.

In the Dataset domain, reliance on non-public datasets was rated as high risk due to limited external verifiability, whereas studies evaluated on established benchmarks such as ImageNet-VID [51,52] or VisDrone-VID [53,65] received low risk ratings. The four high-risk assignments (BViTFPN-TOOD [42], CGFA-Net, SDFMD [41], SST [38]) are specifically tied to the use of custom datasets rather than methodological flaws.

In the Inputs domain, all 21 studies received low risk ratings, as input resolutions, preprocessing pipelines, augmentation strategies, and frame sampling rates were consistently reported.

Evaluation metrics reporting was similarly strong, with 90% studies receiving low risk ratings. The two moderate ratings reflect incomplete efficiency metric reporting rather than inaccuracy (no FPS/FLOPs reported, only parameter count).

In the Methodology and Reproducibility domain, 43% studies received moderate risk ratings. Records penalized in this domain reflect incomplete reporting across one or more sub-criteria, including insufficient architecture description, missing training or inference configuration details, unspecified hardware platforms, inadequate description of temporal modeling mechanisms, and unavailable or conditionally promised source code. Statements such as “code will be made available” were treated as insufficient for reproducibility. Identifying source code often required extensive cross-referencing beyond the manuscript itself.

The Fair Comparison and Generalizability domain yielded moderate risk ratings for five out of 21 studies (24%), primarily where baseline comparisons were conducted under non-equivalent conditions or where efficiency claims were reported in isolation without comparative context against relevant baselines.

Across domains, ratings are strongest for Inputs and Evaluation Metrics, and weakest for Datasets and Methodology and Reproducibility, driven, respectively, by non-public dataset reliance and incomplete reproducibility reporting.

**Figure 3:**
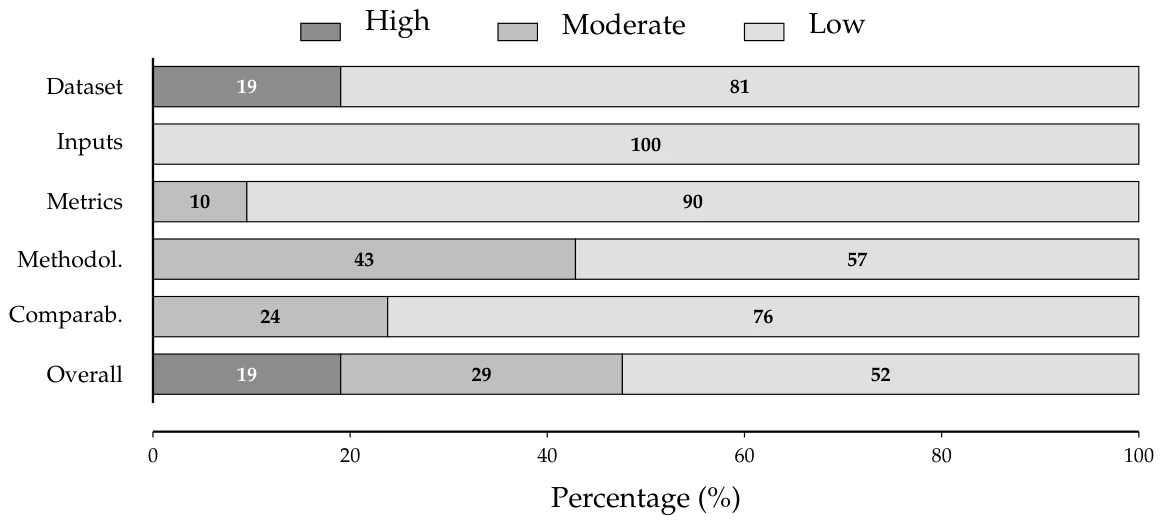
Risk of bias summary plot (*N* = 21). Aggregated risk-of-bias scores across the five domains (Dataset, Inputs, Evaluation Metrics, Methodology and Reproducibility, Fair Comparison and Generalizability).

Applying ROB-CVA’s conservative aggregation rule to the domain ratings above, 11 studies were classified as overall low risk of bias, six as moderate risk, and four as high risk. These figures reflect the review-specific, non-validated ROB-CVA instrument and its conservative aggregation rule, under which a single high-risk domain sets the overall rating (see Section 5.3.3 and the sensitivity analysis in Section 4.5.5). Figure 3 summarizes risk levels across all five domains and overall. Moderate and high ratings are concentrated in the Dataset and Methodology and Reproducibility domains, driven by non-public dataset reliance, incomplete reporting across reproducibility sub-criteria, and conditionally promised code availability.

### 4.4. Results of Individual Studies

The included studies reported a wide range of evaluated performance and efficiency results, reflecting the diversity of datasets, architectures, and evaluation protocols used across VOD literature. Table 7 summarizes the per-study results for all models evaluated on ImageNet-VID, Table 8 covers domain-specific evaluations, while Table 9 covers studies evaluated on heterogeneous datasets. Both tables report primary performance metrics (mAP_50_) alongside available computational characteristics (FPS, latency, and FLOPs).

#### 4.4.1. ImageNet-VID Evaluations

Among the studies evaluated on ImageNet-VID, mAP_50_ ranged from 67.4 (SALISA [31]) to 92.4 (DiffusionVID, Swin-B variant [50]), with the majority of primary corpus entries falling between 80.1 and 90.0. Performance was most frequently reported using mAP_50_, consistent with ImageNet-VID benchmark conventions. Studies such as MaskVD [40], SALISA [31], and QueryProp [46] also reported full COCO-style metrics (AP_*S*_, AP_*M*_, AP_*L*_), providing finer-grained breakdowns not available for the remaining ImageNet-VID entries. mAP_75_ and mAP_50–95_ are also omitted in Table 7 as they are not consistently reported in all studies. Studies operating in specialized domains reported task-specific measures supplementary to mAP, including F1-score, recall, and AUC-PR, reflecting domain evaluation conventions in fall detection and event-based sensing. Notably, for efficiency, all configurations of SALISA [31] report remarkably low FLOPs (G), despite the decreased performance, while FAM (RT-DETR) [39] and FAQ [48] show very competitive latency ranging between 7.5–14.1 ms and 9.2–12.3 ms, respectively.

Hardware platforms varied substantially across studies, limiting direct cross-study comparison. Parameter counts were also not reported consistently across the full corpus. SwinVid [37] did not report FPS, latency, FLOPs, or hardware platform, instead reporting GPU memory usage of 4.12 GB (Swin-T) and 6.94 GB (Swin-S) as its sole computational cost indicator.

#### 4.4.2. Heterogeneous Dataset Evaluations

Results on domain-specific datasets (drones) and heterogeneous datasets are summarized in Table 8 and Table 9. A minority of studies relied on non-public or custom datasets, which precluded external verification of reported results. Studies using EPIC-KITCHENS [66] were excluded from these tables to avoid diluting the analysis with downstream tasks such as video action recognition. Studies using the dataset for primary or supplementary ablation studies as well as any other datasets utilized in this manner were also excluded.

### 4.5. Results of Synthesis

This section presents the structured quantitative and narrative synthesis described in Section 3.9, combining descriptive statistics in accordance with PRISMA.

#### 4.5.1. RQ1—Architectural Categorization Results

To facilitate a structured synthesis, we developed a custom architectural taxonomy consisting of four mutually exclusive categories. This categorization follows the primary architectural distinction each group is known for: detection head formulation for DETR-based and Dense One-Stage models, backbone design for ViTDet-style models, and the relational reasoning mechanism for Graph-based models.

- DETR-based: Employing a transformer decoder with a fixed set of learned object queries to formulate detection as a set-prediction task, supervised via bipartite matching (Hungarian algorithm) to bypass non-maximum suppression (NMS) and anchor design.
- Dense One-Stage: Transformers may enhance the backbone or neck for feature interaction, but detection is performed via dense grid-based or anchor-based prediction heads (e.g., YOLO, SSD, EfficientDet) using fixed spatial assignments.
- ViTDet-style: models using a plain vision transformer backbone with a non-query detection head.
- Graph-based: Detection logic formulated through explicit graph constructions where nodes represent regions or features, using transformer-based attention to operate on relational edges for spatiotemporal or object-to-object reasoning.

**Figure 4:**
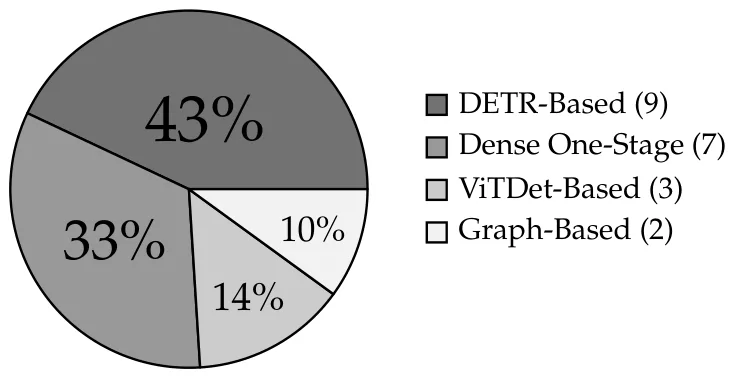
Architectural Distribution (*n* = 21). The legend includes aggregated totals for each bin.

Our categorization revealed DETR-based architectures as the most prominent family in the corpus, representing 43% of the selected studies. As shown in Figure 4, DETR-based architectures appear from 2022 onward within the 2020–2025 study window and constitute the most frequent category. Dense One-Stage detectors account for the second largest portion (33%), reflecting their continued relevance for real-time and resource-constrained applications. Graph-based and ViTDet-based models account for (10%) and (14%), respectively. Collectively, the last two categories illustrate diversification of transformer-based approaches beyond the DETR paradigm, though no strong year-to-year growth pattern is observed within any of these families given the limited sample size.

A notable characteristic across the corpus is the prevalence of complete end-to-end detectors as base architectures. Temporal modeling is integrated as plug-in modules rather than standalone temporal backbones, especially within the DETR-based category. This may reflect a broader tendency toward adapting high-performing image detectors rather than designing video-specific architectures from the ground up, though this tendency is not uniform across all architectural families in the corpus.

#### 4.5.2. RQ2—Temporal Modeling

To address RQ2, the 21 included studies incorporated a diverse range of temporal modeling paradigms, reflecting the breadth of the lightweight transformer VOD design space.

The most populous group consists of studies employing query-based cross-frame aggregation, where temporal information is introduced at the level of object queries within a DETR-style decoder. However, the sub-mechanisms within this group are substantively distinct. FAQ [48] aggregates queries from neighboring frames using cosine similarity in the vanilla aggregation module and also proposes generation and aggregation of dynamic queries through a transformer-based module. QueryProp [46] propagates sparse object queries from key frames to non-key frames via an adaptive propagation gate, avoiding pixel-level feature alignment entirely. TransVOD [47] introduces dedicated temporal transformer encoder and decoder components to fuse spatial and temporal query representations jointly. ClipVID [32] takes a different approach by matching object queries across frames based on learned identity embeddings and aggregating region features from identity-consistent queries via cross-attention. C2FDrone [34] initializes decoder queries from coarse spatial proposals pooled across all frames in a batch, effectively encoding multi-frame priors into the query initialization stage rather than aggregating after decoding. DiffusionVID [50] conditions refined queries on a compact core-set constructed from object query representations sampled across the full video, with cross-attention generating adaptive conditioning vectors that inject video-wide temporal context into each query.

A second group operates at the feature or proposal-level rather than the query level. SwinVid [37] employs SELSA to aggregate proposal-level features across frames based on semantic similarity. YOLOV [67] was the first to propose FAM. FAM (RT-DETR) [39] improves upon FAM and integrates cross-frame self-attention directly within the decoupled localization and classification detection head branches of RT-DETR. Thus, it performs separate self-attention operations on localization and classification features, respectively, with similarity-filtered average pooling over reference features to aggregate temporal context across frames.

A third group exploits temporal redundancy instead of aggregating information across frames. MaskVD [40] applies dynamic masking based on inter-frame redundancy to reduce redundant computation at inference time. Similarly to masking, eventful transformers [49] identify and selectively update only tokens that have changed significantly between frames via a delta-based gating mechanism. SDFMD [41] reduces the temporal volume through spatial-domain filtering instead of explicit cross-frame attention, treating temporal redundancy as a spatial compression problem. Building on this redundancy-suppression paradigm, RGTP [45] introduces a training-free framework that leverages historical attention roll-out and token tracking to prune current input tokens based on their ancestral contribution to previous frame predictions.

Graph-based spatiotemporal reasoning constitutes a further distinct approach. DGC-Net [44] constructs dual-level graphs over frame-level and proposal-level nodes, applying graph transformers and contrastive learning for temporal feature aggregation. EGSST [35] builds spatiotemporal graphs from event streams, utilizing a spatiotemporal sensitivity module (SSM) and an adaptive temporal activation controller (TAC) to mimic human eye perception by selectively modulating temporal attention based on relative dynamics.

Windowed and module-based temporal attention forms another identifiable cluster. TransVisDrone [33] and SwinVid [37] both leverage Swin-style shifted temporal windows to capture local temporal dependencies within a hierarchical feature representation. ST-Trans [36] introduces a dedicated spatiotemporal transformer module for inter-frame alignment in infrared small target detection. CGFA-Net [30] introduces a close-loop edge-based position encoding module, in which an edge-based attention mechanism combines an edge map with a location-possibility map derived from the previous frame’s heatmap. This, in turn, guides position encoding, propagating detected object locations across event frames. TST-YOLOv8 [43] integrates a time–space transformer that applies self-attention jointly across spatial and temporal dimensions within a YOLO-based pipeline.

The final group of studies adopts non-attentional temporal mechanisms. BViTFPN-TOOD [42] maintains a recurrent token state across frames via block-recurrent feature pyramids, enabling feature reuse without explicit cross-frame attention. SALISA [31] replaces temporal attention with saliency-based input sampling, selectively routing informative frames into the detection pipeline. SST [38] employs spiking neural networks to process event streams, encoding temporal dynamics through spike timing rather than learned attention weights.

**Figure 5:**
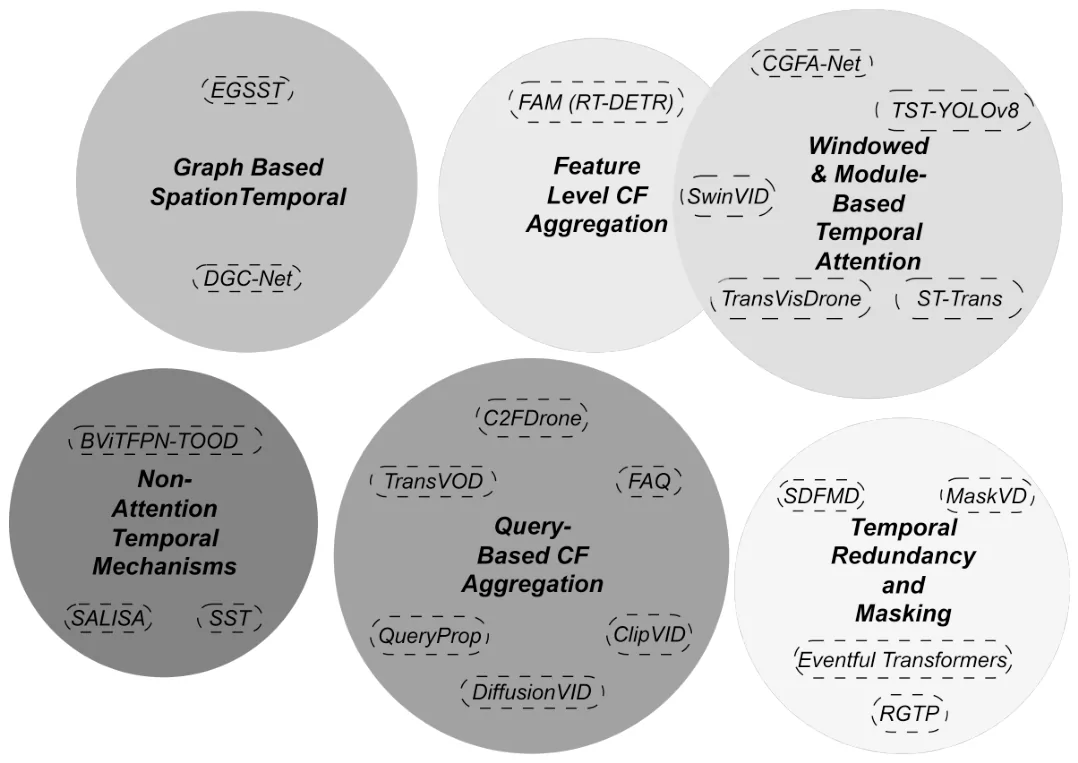
Qualitative analysis of temporal modeling approaches groups. Distinct gray shading is used to differentiate functional categories. Dotted ovals represent individual studies. CF = Cross Frame.

In summary, these mechanisms (see Figure 5) span a broad design space from explicit cross-frame query propagation to implicit redundancy suppression and biologically-inspired spiking representations. Several studies combine multiple strategies, and temporal components appear across all architectural bins defined in RQ1. The limited corpus size cannot support the extraction of clear patterns in regards to dominant temporal modeling paradigms. We conclude that the frequency of specific mechanisms should not be taken in isolation but considered together with the quantifiable metrics in each category as well as the diverse deployment contexts in the corpus, which place varying requirements on what constitutes adequate temporal reasoning under efficiency constraints.

#### 4.5.3. RQ3—Optimization Strategies

Efficiency across the corpus was achieved through a combination of three recurring strategies: structural simplification, attention-scope reduction, and temporal-volume minimization. These strategies are not mutually exclusive, and most studies combined two or more.

Structural simplification was the most pervasive strategy, achieved through compact convolutional blocks, hybrid CNN-Transformer pipelines, or mobile-grade backbones (e.g., [42]), maintaining representation power at reduced computational cost.

Attention-scope reduction constrained the quadratic cost of global self-attention through techniques such as Swin-style shifted windows (TransVisDrone, SwinVid [33,37]) or dynamic redundancy-aware masking (MaskVD [40], eventful transformers [49]).

Temporal-volume minimization reduced the amount of temporal information processed per inference step, through saliency-based frame selection (SALISA [31]), spatial-domain redundancy filtering (SDFMD [41]), recurrent feature reuse (BViTFPN-TOOD [42]), or clip-level query aggregation (ClipVID [32]).

#### 4.5.4. RQ4—Dataset-Specific Insights

The diversity of evaluation datasets described in Section Corpus-reported Evaluation Datasets strongly influenced the comparability of reported results across the corpus. ImageNet-VID was the dominant benchmark, used by 57% (12/21) of included studies, and its established evaluation conventions enabled a relatively consistent basis for comparison in terms of metric consistency and reporting completeness. The adoption of drone-specific datasets (e.g., VisDrone-VID, UA-DETRAC) highlights how genuine contributions are often diffused in domain-focused works and downstream vision tasks, complicating cross-domain synthesis. Studies relying on custom or non-public datasets showed greater variability in performance. Thus, interpreting performance and efficiency trade-offs with only author-used baselines was challenging.

Even within ImageNet-VID, variations in backbone, input resolution, number of frames, inference settings, hardware, and evaluation protocols persist. As a result, a descriptive ${\mathrm{mAP}}_{50}$ range of 67.4–92.4 cannot provide a meaningful ranking of model quality. Nevertheless, through reported efficiency metrics, some trade-offs are observed even without cross-study comparison. Namely, SALISA [31] reported low FLOPs at the cost of reduced accuracy, while FAM (RT-DETR) [39] balanced latency with competitive accuracy.

#### 4.5.5. Sensitivity Analysis

To assess the robustness of the reported risk-of-bias distribution based on our aggregation rule choice, a post hoc sensitivity check was conducted using a majority-rule alternative. In this alternative rule, overall risk is rated High only if at least two of five domains are rated High. Under this alternative rule, no study meets the threshold for an overall High rating. The four studies rated High under the conservative rule are instead rated Moderate. Five studies with a single Moderate ranking across the five domains, also move from Moderate to Low. The resulting distribution is 0% High, 24% Moderate, 76% Low, compared with 19% High, 29% Moderate, 52% Low under the conservative rule reported in Section 4.3 in our review-specific, non-validated ROB-CVA framework. The reported risk distribution is therefore sensitive to the choice of aggregation rule.

### 4.6. Reporting Biases

Reporting patterns across included studies reflect several bias categories identified a priori in Section 3.7.

#### 4.6.1. Dataset and Outcome Reporting

Dataset selection bias was concentrated among application-driven studies relying on non-public or domain-specific datasets, limiting external verification. Rather than systematic reporting bias, omissions appear driven by heterogeneous publication practices across venues and application domains. Overall, no systematic pattern of selective outcome reporting was identified.

#### 4.6.2. Reproducibility and Code Availability

Five studies did not provide full source code. Two studies are marked as “available upon request” or express intent to share later. Missing hyper-parameter settings, incomplete training descriptions, and absent evaluation details were also observed, particularly among application-driven studies. These limitations restrict the ability to verify results or perform fair cross-model comparisons, even when the underlying datasets are public. Reproducibility bias manifested primarily through missing code availability and incomplete training configurations rather than architectural under-reporting, which was generally adequate.

#### 4.6.3. Hardware Reporting and Evaluation Metrics

Hardware-dependent performance bias was the most pervasive reporting bias identified. Benchmarking platforms ranged from resource-constrained embedded devices (Jetson Xavier) to high-end GPUs (RTX 3090) and data-center-grade hardware (V100, A100), making direct cross-study efficiency comparison unreliable. Only two studies, C2FDrone [34] and TransVisDrone [33], reported cross-platform evaluations, which represent the strongest available benchmarking evidence for deployment generalization. Metric reporting bias was evident in the fragmented use of efficiency indicators, displaying an overall lack of standardization across included studies. Most studies reported inference speed in either FPS or latency in ms. Fewer than half provided a complete computational profile including latency, FLOPs, and parameter counts, while power consumption was rarely reported.

### 4.7. Certainty of Evidence

As noted in Section 3.11, a conventional GRADE assessment was not applied. Instead, certainty was assessed qualitatively from the ROB-CVA risk-of-bias distribution reported in Section 4.3, with each of its domains contributing equally. With 52% of studies classified as low risk under the review-specific, non-validated ROB-CVA framework, this majority is insufficient to support an unqualified low-risk rating for the evidence base as a whole. The moderate-risk share (29%) is substantial and is not treated as negligible relative to the low-risk majority. The overall certainty of evidence is therefore rated low to moderate. This rating is supported by the variability in benchmark datasets, evaluation protocols, and efficiency metric reporting, which limit the generalizability of individual study findings. Reproducibility concerns, including unavailable source code and incomplete training configurations, further constrain independent verification of reported results. Meaningful cross-study comparison is restricted to the subset of studies sharing common benchmarks, primarily ImageNet-VID, where evaluation conventions are sufficiently standardized to support cautious quantitative observation. Conclusions drawn from this review should be interpreted accordingly, with caution applied to claims reported on heterogeneous or non-public datasets.

## 5. Discussion

### 5.1. Interpretation

The included studies present a picture of an active but fragmented landscape, shaped by efficiency constraints, evolving architectural trends, and inconsistent evaluation practices. Within the included studies, hybrid architectures are more prevalent than traditional spatiotemporal approaches such as optical flow or heavy temporal modules. DETR-based architectures clearly dominate the corpus (9/21, 43%), with Dense One-Stage detectors second (7/21, 33%), suggesting that transformers function more commonly as specialized head or encoder enhancements within established detection paradigms than as full-scale architectural replacements.

Among the included studies, efficient temporal aggregation is more commonly adopted than dense volumetric reasoning. Models such as FAM (RT-DETR) [39] illustrate this tendency, achieving competitive accuracy and latency on ImageNet-VID through streamlined detection-head-level aggregation rather than heavy spatiotemporal processing. DiffusionVID [50] demonstrates that significant accuracy gains are achievable within this paradigm, attaining over 92% mAP_50_ on ImageNet-VID with competitive latency. TransVOD [47] remains a seminal contribution in this space, introducing dedicated temporal transformer encoder and decoder components that set a foundational reference for subsequent query-based temporal aggregation approaches. SALISA [31] occupies a different point on the efficiency frontier, reporting the lowest FLOPs on the ImageNet-VID subset (0.86 G, 3-frame sampling) at the cost of reduced accuracy, demonstrating the breadth of the accuracy–efficiency trade-off space within the corpus. This preference for temporal streamlining is a characteristic feature of the included studies; only 2 out of 21 studies, DGC-Net [44], and EGSST [35], adopt explicit graph-based temporal reasoning, while the majority integrate temporal context through comparatively lighter alternatives.

The interpreted corpus indicates how the tension between transformer design and computational overhead is being navigated in research. Video-specific logic is not abandoned but instead refined through methods like the shifted temporal windows seen in SwinVid [37]. This allows the models to exploit necessary inter-frame dependencies while remaining viable for real-time edge deployment.

### 5.2. Limitations of Evidence

Several structural limitations in the evidence base constrain the interpretability and generalizability of the synthesis presented here. This review includes studies that propose, measure, and report a quantifiable reduction in computational cost relative to a baseline, as defined in Table 2, rather than studies merely self-described as lightweight. The limitations below describe how this evidence base, even when correctly scoped, remains difficult to compare and interpret as a whole.

#### 5.2.1. Comparability of Efficiency Claims

Efficiency claims across the corpus were not sufficiently quantified in a form that permits meaningful cross-study comparison, using the inconsistent metrics noted in Section 1.2. There is no shared reference point to compare these gains. A further complication is the diffusion of advances into downstream and domain-specific tasks, as shown in Table 9. Evaluation conventions in these tasks diverge from the general VOD literature. This makes it hard to distinguish whether reported efficiency gains reflect real architectural progress or simply the characteristics of the specific task and dataset.

#### 5.2.2. Benchmarking, Baseline Selection, and Hardware Comparability

A further constraint on cross-study synthesis concerns the selection of comparison points against which efficiency and accuracy claims are made. Benchmarking in this field is author-driven: each study selects its own baselines relying on scientific intuition rather than external enforcement of baseline relevance, recency, or completeness and then evaluates both the baselines and proposals. While this practice is individually reasonable, since authors are best positioned to select baselines relevant to their specific architecture, it emphasizes the difficulty of synthesis in a systematic review.

This issue is independent of, yet exacerbated by, the hardware heterogeneity already discussed in Section 4.6. Original authors are required to contextualize their proposals within a unified evaluation framework, effectively shifting the burden of quantitative validation onto them.

Full hardware reporting is therefore necessary as without it, efficiency metrics cannot be interpreted. Without disclosed hardware specifications, metrics like FPS or latency become unanchored. Furthermore, without a hardware-normalized reference, FLOPs comparisons are the closest available proxy. However, FLOPs understate the true computational cost of attention mechanisms, which introduce memory bandwidth and cache overhead not reflected in operation counts alone. FLOPs therefore reflect theoretical computational complexity, whereas latency and FPS reflect measured inference efficiency under a specific hardware and software configuration. The two should be read as distinct, complementary indicators instead of substitutes for one another. This is particularly relevant given that attention-based temporal modules are a central architectural component across the corpus. Efficiency comparisons in this field are therefore best interpreted as comparisons against author-selected, often hardware-specific reference points, rather than as comparisons against a stable, field-wide standard.

#### 5.2.3. Dataset Selection

Benchmark diversity has become a significant obstacle to meaningful cross-study comparison. As described in Section 4.2 and Section 4.4, while ImageNet-VID remains the dominant shared benchmark (12 of 21 studies), a substantial portion of the evidence has migrated toward niche domain-specific datasets. This fragmentation prevents any emergence of a unified state-of-the-art ranking and suggests that VOD as a vision task is being increasingly superseded through application-specific sub-tasks. Although domain-specific evaluation is scientifically legitimate, the absence of a supplementary evaluation on a broader benchmark limits recognition of advances that generalize beyond the intended application. As a result, comparative assessment across the included studies becomes structurally difficult.

#### 5.2.4. Frame-by-Frame Inference as an Alternative Lightweight Paradigm

The 20 studies excluded from the corpus under the Temporal Modeling criterion (see Appendix B, Table A2) represent more than a methodological boundary. Saliency-based, stateless, and frame-by-frame approaches constitute a philosophically distinct but legitimate conception of lightweight VOD, one that achieves efficiency precisely by avoiding the overhead of cross-frame information exchange. Rather than treating temporal reasoning as a prerequisite for deployment, these architectures treat each frame as an independent inference problem, accepting a trade-off between temporal coherence and computational simplicity. This design philosophy is not inferior to explicit temporal modeling but rather a different response to the same efficiency constraint. The decision to exclude these studies reflects the specific focus of this review on architectures that exploit video’s temporal structure, not a judgment on the validity of stateless approaches. Future work synthesizing the full spectrum of lightweight VOD, including both temporal and stateless paradigms, would provide a more complete picture of the design space and the conditions under which each philosophy is preferable.

### 5.3. Limitations of Review Process

#### 5.3.1. Search and Retrieval

The formal search relied exclusively on indexed peer-reviewed publications and no preprint archives such as arXiv. Peer review provides a baseline check on methodological quality that preprints do not have. Preprint-first work becomes eligible for retrieval once published in a peer-reviewed venue and indexed in one of the five searched databases. This limitation is common in rapidly evolving fields such as AI and CV, where high-impact studies often appear first on preprint servers that are not indexed in the selected databases. To further strengthen search coverage, backward citation tracking (BCT) was conducted on the reference lists of the primary corpus. Forward citation tracking (FCT) was not performed, as the included studies span publication years up to 2025 and forward citation data would be limited and likely incomplete for the most recent entries. The lack of FCT may favor the selection of older and established work and may miss emerging studies citing the included papers. No distinct screening, extraction, or risk-of-bias procedure was applied to backward-citation-tracked studies relative to the primary-search corpus.

Regarding the search phase, specific database indexing tool limitations encountered were disclosed in Section 3. The unified search string was designed to maximize reproducibility across databases, with minimal platform-specific adjustments. This approach prioritizes consistency over precision, accepting a higher rate of irrelevant records at the identification stage in exchange for a transparent and replicable retrieval process. Similarly, the literature search concluded on 28 August 2025, and studies published or made available after this date are not captured by the review.

The search string was constructed to correspond one-to-one with the review’s intersecting topics. Individual keyword choices may not capture all terminology authors use for efficiency gains. Studies using non-matching language could have been missed at retrieval, rather than excluded later at screening. An alternative design would retrieve a broader set of records with a simpler string, applying the efficiency criterion during title/abstract and full-text screening instead. This trade-off between retrieval-stage precision and screening-stage burden was examined using preliminary search strings to avoid the cost of a substantially larger set of retrieved records. However, this choice may still have resulted in missed coverage.

A formal validation of the search strategy against a known set of seminal or independently established eligible works was not performed. Identifying such a set within a scope as narrow as this review’s intersection of criteria is challenging without the selection itself being informed to any degree by the search results already obtained. Given this risk of circularity, no retrospective recall test was conducted, and the completeness of the search strategy therefore remains partially unverified beyond the safeguards provided by backward citation tracking.

#### 5.3.2. Corpus Size and Scope

The small number of included studies (*n* = 21, 22 reports, after BCT) is an inherent consequence of the narrow thematic intersection studied. The large initial corpus (*n* = 1343) and high exclusion rate attributable to specific eligibility criteria are consistent with this. Our strict definition of transformer architectures (see Table 2) may have excluded some borderline cases (e.g., hybrid models with partial transformer components). This definition keeps the review’s focus on transformer encoder/decoder stacks, as defined in Vaswani et al. [26]. Readers seeking a broader synthesis of transformer-based detection or lightweight deep learning are directed to the complementary surveys listed in Table 1.

A similar scope decision applies to the Efficiency criterion (Table 2), which accepts any of parameter count, inference speed, or FLOPs as evidence of a reduction in computational cost without requiring a specific metric. These metrics are not interchangeable. A lower parameter count does not guarantee lower latency. A lower FLOPs count does not guarantee lower memory-bandwidth cost, since attention operations carry overhead that FLOPs do not capture. As a result, studies admitted under this single criterion are not necessarily efficient in the same way. This heterogeneity comes from the criterion’s scope, not only from inconsistent reporting. This is a further reason this review relies on narrative rather than pooled synthesis.

#### 5.3.3. Risk-of-Bias and Certainty of Evidence

ROB-CVA, proposed and applied first in this review, has not been externally validated. Its conservative aggregation rule may overstate risk relative to less conservative alternatives. A sensitivity analysis using a majority-rule alternative is reported in Section 4.5.5. The reported risk distribution is therefore sensitive to the choice of aggregation rule, while the conservative rule used is better interpreted as an upper bound on risk, not a validated estimate. Currently, all four studies rated High overall are driven by a High rating in the Dataset domain, reflecting reliance on non-public datasets. While a non-public dataset limits external verification and reproducibility, it does not necessarily indicate methodological flaws that a ROB assessment is intended to capture. The Dataset domain’s public-availability criterion currently conflates these two concerns within a single rating.

Additionally, formal inter-rater agreement statistics for study screening and risk-of-bias assessment are not reported. All stages involved two independent reviewers with disagreements resolved through discussion, but per-reviewer decision records sufficient to compute a statistic (e.g., Cohen’s kappa) were not retained. The reporting bias assessment was qualitative, based on comparing methods and results sections within each study to identify selective reporting or presentation of data.

#### 5.3.4. Data Extraction and Heterogeneity

A limitation encountered was the heterogeneity of performance reporting. The process of compiling metrics in Table 7, Table 8 and Table 9 required interpreting mAP thresholds, across several datasets and hardware configurations. While every effort was made to correctly extract and present available data, the lack of raw data for many studies introduces a margin of error in these comparisons. Conversion of compatible metrics for latency was not implemented to avoid compounding homogenization errors.

Future application of this review’s methodology would benefit from pre-specifying extraction and estimation procedures for these cases. Values affected by either exception are not retained in the primary synthesis tables (Table 8 and Table 9) and are instead reported as not reported (NR). This affects one graph-approximated entry (SDFMD [41]) and three IoU-threshold-inferred entries (ST-Trans [36]; TST-YOLOv8 [43] in two datasets). These values are retained, labeled as reviewer-derived, and excluded from the primary synthesis, in Appendix B Table A3. None of the affected values are used in any comparative or ranked claim in this review. Lastly, the heterogeneity of evaluation datasets and metrics could be partially attributed to borderline inclusion studies, thus, reiterating the difficulty in capturing genuine advancements within several disciplines.

### 5.4. Future Directions

Future research should focus on balancing architectural innovation with practical deployability. Promising directions include efficient temporal modules, dynamic token routing, and hybrid designs that leverage both convolutional and transformer-based strengths. Rather than relying solely on the minimal temporal aggregation logic seen in current lightweight designs such as FAM (RT-DETR) [39], future work should prioritize the development of more robust, low-cost temporal modules, drawing on the identity-consistent cross-attention seen in ClipVID [32] or the query propagation strategies in TransVOD [47] and DiffusionVID [50] to ensure that lightweight VOD exhibits genuine spatiotemporal reasoning without prohibitive overhead.

The field would benefit considerably from community-agreed minimum reporting standards. This includes a standard reference device, or at minimum, a small set of representative reference devices spanning edge and data-center tiers, for efficiency evaluation. For datasets, supplementary evaluations on a common general purpose benchmark where technically appropriate, while retaining domain-specific evaluations as primary evaluations, could also aid in connecting related tasks with advancements. Clearer conventions around baseline selection should be set, so that claims are anchored to a comparable, ideally externally curated, set of reference methods rather than to author-driven baselines.

Moreover, to address the interpretive challenges posed by hardware-dependent reporting (ranging from A100 to Jetson Xavier), the community should strive for hardware-agnostic metrics. This would allow for a fairer comparison of architectural efficiency regardless of the deployment platform. The creation of clear taxonomies of inference and training hardware to standardize evaluations and aid in literature synthesis of future works could significantly address this challenge.

Based on the reporting gaps and the 67% (14/21) code availability identified in this review, we propose that future publications adhere to a minimum efficiency profile reporting that includes latency, parameter count, FLOPs, and peak memory usage, alongside clear definitions of mAP thresholds to standardize metadata. For studies with edge deployment as primary scope, the profile should additionally include power consumption metrics. Similar standardized approaches are seen in adjacent computer vision tasks, such as multi-object tracking, where the use of TrackEval [68] is widely adopted in relevant publications. Beyond efficiency metrics, future extraction protocols in this area should also pre-specify input resolution, frame sampling protocol, and evaluation-time batch size as standard fields, given their material influence on the reported efficiency–accuracy trade-off.

Finally, given the identified dataset fragmentation and the migration of standard benchmarks to less accessible platforms, there is an emerging need for new, high-diversity VOD datasets that emphasize video-centric nuances such as long-term occlusions and motion blur, as well as high density annotations across frames. A step in the right direction is taken by studies that incorporated datasets such as UA-DETRAC that attempt to address this gap.

Future work aiming to use methods proposed in this review should prioritize external validation of ROB-CVA. This includes inter-rater reliability testing and application by reviewers involved in broader computer vision. A second direction is refining the Dataset domain criteria to distinguish risk of bias arising from dataset selection from the separate concerns of applicability and external reproducibility that non-public datasets raise, instead of rating both under a single criterion. Both directions are necessary for establishing it as a reusable assessment instrument. Future reviews in this area could also strengthen search validation by pre-specifying a known-item recall test. The test should feature a set of seminal or independently nominated eligible works defined prior to running the search, allowing the final search strings to be evaluated against this reference set without risk of circular selection. Future work should also pursue a deeper cross-study synthesis of the relationship between temporal modeling strategy and efficiency–accuracy trade-offs, building on the standardized reporting practices proposed above to enable more direct comparison than a narrative synthesis allows. Lastly, future synthesis with related scope could propose improved taxonomies for VOD models based on their efficient design paradigms and temporal-reasoning capabilities. These taxonomies could then more effectively map architectural approaches to deployment contexts, given the proposed standardized evaluation conditions improve comparability in the future.

## 6. Conclusions

This study characterizes a nexus of four active research threads, transformer architectures, video object detection, temporal modeling, and efficiency-driven design, that had not previously been jointly synthesized. Across 22 reports and 21 studies meeting strict eligibility criteria, 16 identified through systematic database search and 5 through backward citation tracking, a coherent picture emerges of a field navigating genuine efficiency constraints through architectural advancements, while leaving several methodological questions unresolved.

The most consequential finding of this review is methodological. The included studies reflect a growing body of transformer-based VOD work that claims both efficiency and temporal reasoning, yet lacks the shared benchmarks, reporting conventions, and hardware transparency necessary to evaluate these claims comparatively. A minimum reporting set: latency, parameter count, FLOPs, peak memory, and especially power use for edge applications, measured against a reference hardware set, is proposed toward addressing this gap.

This review adapts PROBAST-AI into ROB-CVA, a structured risk-of-bias framework tailored to efficiency and performance claims in computer vision. ROB-CVA is, to the best of our knowledge, the first application of such a framework within video object detection synthesis. Its application across the full corpus of 21 studies indicates that a majority of studies (52%) were classified as low risk of bias using our review-specific and not externally validated ROB-CVA framework (Section 5.3.3), with the remainder distributed across moderate and high risk under ROB-CVA’s conservative aggregation rule. By introducing a systematic bias assessment, this work provides a rigorous foundation for evaluating lightweight performance claims and contributing to future methodological standards in the field. While ROB-CVA offers a structured and reproducible basis for assessing bias in efficiency-oriented computer vision research, it is not without limitations. Refinement of its domains and criteria, informed by broader application across CV sub-fields beyond VOD, remains a valuable direction for future methodological work.

Architectural mechanisms examined in the corpus reveal a hybridization pattern. Transformers are more commonly deployed as specialist enhancement modules within DETR-based or Dense One-Stage pipelines than as standalone architectural replacements for convolutional approaches. A further observation from the included studies is that temporal modeling strategies tend toward simplification, ranging from full spatiotemporal reasoning toward lightweight redundancy masking and multi-frame query aggregation. Whether this simplification reflects genuine progress toward efficient video handling or a deferral of the harder problem of robust temporal reasoning remains an open question that requires the shared benchmarks and standardized reporting practices called for by this review.

Looking forward, while this review has focused on transformer architectures, the examined efficiency and accuracy trade-offs may also be addressed through alternative sequence modeling paradigms not presented here. The emergence of state space models [69] and state space duality [70] suggests a trajectory in which the broader transformer designs might be achieved at linear-time complexity. When coupled with the efficient attention variants and hybrid CNN-Transformer pipelines identified in this synthesis, these developments offer promising directions for powerful temporal reasoning under edge deployment constraints. Whether these directions produce genuinely efficient VOD advancements, rather than benchmark-optimized architectures, will depend on the community’s adoption of stricter evaluation standards and transparency practices.

## Figures and Tables

**Table 2:** Study Inclusion Criteria.

Category	Criteria
Task	Video object detection.
Architecture	Transformer-based *: at least one complete transformer encoder or decoder stack comprising multi-head self-attention and a position-wise feed-forward network with residual connections and layer normalization [26].
Temporal Modeling	Explicit temporal modeling ^†^ over multiple video frames.
Efficiency	Proposes or evaluates a lightweight ^‡^ architecture or mechanism.
Evidence	Reports quantitative performance metrics (e.g., mAP, FLOPs, parameters, or latency).
Research Scope	Addresses ≥2 of the defined research questions.
Publication	Peer-reviewed journal or conference proceedings, 2020–2025, English language.

* Studies employing isolated attention mechanisms, non-local blocks, relation networks, or set-based prediction heads without a full encoder or decoder stack are excluded. ^†^ Defined as any mechanism that operates over information from multiple frames to reason about or exploit temporal structure, including but not limited to recurrent modeling, sequential feature propagation, cross-frame attention, masked temporal prediction, and memory-based aggregation. ^‡^ Satisfied when a reduction in computational cost is proposed, measured, and reported or when the study targets explicit design for resource-constrained deployment. Comparative metrics (e.g., FLOPs, parameters, or latency) against a baseline must be provided.

**Table 3:** Search keywords grouped by category, including synonyms piloted but excluded due to redundancy or excessive noise. The asterisk (*) wildcard is used to match strings with the attached prefix or suffix string. Core terms were retained in the final search queries.

Category	Search Terms
Lightweight/Efficiency	Core: (lightweight OR efficient OR real-time OR edge)
Video/Temporal Aspects	Core: (video OR stream* OR sequence OR temporal) Considered but excluded: (video analysis OR video analytics OR video surveillance)
Object Detection	Core: (object detection OR object recognition) Considered but excluded: (visual detection OR target detection OR instance detection)
Deep Learning Architecture	Core: (transformer* OR ViT)

**Table 4:** ROB-CVA tool adapted from PROBAST-AI criteria and used for efficient transformer-based video object detection with temporal modeling in this review.

Domain	ROB-CVA Criteria
Datasets	– Dataset publicly available and accessible without restriction. – Dataset is sufficiently diverse to support generalizability claims without introducing algorithmic bias.
Inputs	– Input resolutions, preprocessing, augmentations, and modality-specific parameters reported.
Evaluation Metrics	– Explicit and standard accuracy metrics appropriate for the task are used. – Metrics defined and applied consistently across datasets and baselines.
Methodology and Reproducibility	– Architecture described in sufficient detail to allow reproduction. – Hardware platform specified. – Training and inference configurations and hyper-parameters reported or supplemented through source code. – Source code or a pretrained model publicly available at time of publication.
Fair Comparison and Generalizability	– Fair selection of baselines. – Baseline comparisons conducted under equivalent and controlled conditions. – Accuracy and efficiency claims compared against relevant baselines. – Dataset appropriate for the claims made.

**Table 5:** Overview of included studies, sorted by year within each group. Primary benchmark datasets only; ablation-only datasets and other vision task datasets are excluded from this synthesis. Code refers to code availability based on extracted information.

Model	Year	Domain	Code	Benchmark Dataset(s)
CGFA-Net [30]	2022	Neuromorphic/Event	N	EventKITTI, KITTI
SALISA [31]	2022	Fundamental VOD	N	ImageNet-VID, UA-DETRAC
ClipVID [32]	2023	Fundamental VOD	A	ImageNet-VID
TransVisDrone [33]	2023	UAV/Aerial	A	FL-Drones, NPS-Drones
C2FDrone [34]	2024	UAV/Aerial	U	FL-Drones, NPS-Drones
EGSST [35]	2024	Neuromorphic/Event	A	Gen1, 1-Megapixel
ST-Trans [36]	2024	Thermal/Infrared	A	SIRSTD
SwinVid [37]	2024	Fundamental VOD	A	ImageNet-VID
SST [38]	2024	UAV/Aerial	N	Custom event stream dataset
FAM (RT-DETR) [39]	2025	Fundamental VOD	A	ImageNet-VID
MaskVD [40]	2025	Fundamental VOD	A	ImageNet-VID, KITTI
SDFMD [41]	2025	Fundamental VOD	N	Custom video dataset
BViTFPN-TOOD [42]	2025	Thermal/Infrared	U	Custom thermal video dataset
TST-YOLOv8 [43]	2025	Fall Detection	N	CUCAFall, DiverseFALL10500
DGC-Net [44]	2025	Fundamental VOD	A	ImageNet-VID
RGTP [45]	2025	Fundamental VOD	A	ImageNet-VID
Studies identified via backward citation tracking (BCT)				
QueryProp [46]	2022	Fundamental VOD	A	ImageNet-VID
TransVOD [47]	2022	Fundamental VOD	A	ImageNet-VID
FAQ [48]	2023	Fundamental VOD	A	ImageNet-VID
Eventful Transformers [49]	2023	Fundamental VOD	A	ImageNet-VID
DiffusionVID [50]	2023	Fundamental VOD	A	ImageNet-VID

Note: Code Availability abbreviations: A = Available, U = Upon Request/Upcoming, N = Not Available.

**Table 6:** Traffic-lights table of the RoB assessment for all included studies alphabetically. Categories in full are Dataset, Inputs, Evaluation Metrics, Methodology and Reproducibility, Fair Comparison and Generalizability, and Overall.

Model	Dataset	Inputs	Eval. Metrics	Method. and Repro.	Fair Comp. and Gen.	Overall
BViTFPN-TOOD [42]	H	L	L	M	M	H
C2FDrone [34]	L	L	L	M	L	M
CGFA-Net [30]	H	L	L	M	M	H
ClipVID [32]	L	L	L	L	L	L
DGC-Net [44]	L	L	L	L	L	L
EGSST [35]	L	L	L	L	L	L
MaskVD [40]	L	L	L	L	L	L
FAM (RT-DETR) [39]	L	L	L	L	L	L
SALISA [31]	L	L	L	M	L	M
SDFMD [41]	H	L	L	M	M	H
SST [38]	H	L	M	M	M	H
ST-Trans [36]	L	L	L	L	L	L
SwinVid [37]	L	L	M	L	L	M
TransVisDrone [33]	L	L	L	L	L	L
TST-YOLOv8 [43]	L	L	L	M	L	M
RGTP [45]	L	L	L	M	L	M
Studies identified via backward citation tracking (BCT)						
DiffusionVID [50]	L	L	L	L	L	L
Eventful Transformers [49]	L	L	L	M	M	M
FAQ [48]	L	L	L	L	L	L
QueryProp [46]	L	L	L	L	L	L
TransVOD [47]	L	L	L	L	L	L

Legend: L = Low Risk (light grey), M = Moderate Risk (medium grey), H = High Risk (dark grey), per domain, as defined in Table 4. Overall risk is derived using the conservative aggregation rule specified in Section 3.7: High if at least one domain is High; Moderate if at least one domain is Moderate and none are High; Low only when all domains are Low.

**Table 7:** Performance and efficiency metrics of included studies evaluated on ImageNet-VID sorted alphabetically by model name within each group. Speed/latency reported in their original metrics (ms/FPS) to avoid homogenization errors. Comparisons across studies are descriptive as opposed to comparative, given differing hardware platforms and evaluation conditions. Similarly, speed values are not intended for direct cross-study ranking.

Model	Arch.	Backbone	Hardware	mAP_50_	Speed	FLOPs (G)
ClipVID [32]	DETR-based	ResNet-101	—	84.7	39.3 fps	—
ClipVID [32]	DETR-based	ResNeXt-101	—	85.8	25.1 fps	—
DGC-Net [44]	Graph-based	ResNet-101	RTX 3090	86.3	90.2 ms	—
DGC-Net Lite [44]	Graph-based	ResNet-101	RTX 3090	85.1	24.9 ms	—
DGC-Net [44]	Graph-based	ResNeXt-101	RTX 3090	87.3	191.5 ms	—
DGC-Net Lite [44]	Graph-based	ResNeXt-101	RTX 3090	86.2	38.7 ms	—
MaskVD [40]	ViTDet-based	ViT-B	RTX A6000	82.11	42.15 ms	106.43 ^⋄^
FAM (RT-DETR) [39]	DETR-based	ResNet-18	A800	73.2	7.5 ms	63.6
FAM (RT-DETR) [39]	DETR-based	ResNet-34	A800	76.1	9.4 ms	96.5
FAM (RT-DETR) [39]	DETR-based	ResNet-50	A800	84.8	11.8 ms	142.2
FAM (RT-DETR) [39]	DETR-based	ResNet-101	A800	90.0	14.1 ms	265
RGTP [45]	ViTDet-Based	ViT-B	A5000	82.09	27 ms	46.78
SALISA [31]	Dense One-Stage	EfficientNet-B2	Tesla V100	74.5	—	7.2
SALISA [31]	Dense One-Stage	EfficientNet-B3	Tesla V100	75.4	—	14.4
SALISA [31]	Dense One-Stage	EfficientNet-B0 ^‖^	Tesla V100	67.4	—	0.86
SALISA [31]	Dense One-Stage	EfficientNet-B0	Tesla V100	68.7	—	1.5
SALISA [31]	Dense One-Stage	EfficientNet-B1	Tesla V100	71.8	—	3.38
SwinVid [37]	Dense One-Stage	Swin-T	—	80.1	—	—
SwinVid [37]	Dense One-Stage	Swin-S	—	84.3	—	—
Studies identified via backward citation tracking (BCT)						
DiffusionVID [50]	DETR-based	ResNet-101	RTX 3090	86.9	21.5 ms	—
DiffusionVID [50]	DETR-based	Swin-B	RTX 3090	92.4	37.0 ms	—
Eventful Transformers ^§^ [49]	ViTDet-based	ViT-B	RTX 3090	81.25	—	122.3
FAQ (SMCA-DETR) [48]	DETR-based	ResNet-50	Tesla A100	79.1	10.9 fps	—
FAQ (Conditional-DETR) [48]	DETR-based	ResNet-50	Tesla A100	79.2	10.3 fps	—
FAQ (DAB-DETR) [48]	DETR-based	ResNet-50	Tesla A100	79	9.2 fps	—
FAQ (Deformable-DETR) [48]	DETR-based	ResNet-50	Tesla A100	81.7	12.3 fps	—
QueryProp [46]	DETR-based	ResNet-101	TITAN RTX	82.3	32.5/26.8 fps ^¶^	—
QueryProp [46]	DETR-based	ResNet-50	TITAN RTX	80.3	45.6/36.8 fps ^¶^	—
TransVOD [47]	DETR-based	Swin-T	Tesla V100	83.7	29.6 fps	—

^⋄^ Originally reported as GMACs; retained as approximate GFLOPs equivalent. ^§^ Tunable method; value shown corresponds to author-highlighted operating point (r = 768). ^¶^ Online/offline FPS, respectively. ^‖^ Every 3 frames.

**Table 8:** Performance and efficiency metrics for models evaluated on drone-specific and traffic datasets. Speed and latency reported in their original units (FPS and ms, respectively) to avoid compounding homogenization errors. Comparisons across studies are descriptive as opposed to comparative, given differing hardware platforms and evaluation conditions. Similarly, speed values are not intended for direct cross-study ranking.

Model	Architecture	Backbone	Hardware	mAP_50_	Speed	FLOPs
FL-Drones						
C2FDrone [34]	DETR-based	Swin-B	RTX A6000	84.0	>35 fps	—
TransVisDrone [33]	DETR-based	CSPDarknet	A6000/Xav.	75.0	24.6 fps	—
NPS-Drones						
C2FDrone [34]	DETR-based	Swin-B	Jetson Xavier	93.0	31 fps	—
TransVisDrone [33]	DETR-based	CSPDarknet	RTX A6000	95.0	24.6 fps	—
VisDrone-VID2019						
DGC-Net [44]	Graph-based	ResNet-101	RTX 3090	61.5	—	—
UA-DETRAC						
SALISA [31]	Dense One-Stage	EfficientNet-B2	Tesla V100	—	—	5.9
SALISA [31]	Dense One-Stage	EfficientNet-B3	Tesla V100	—	—	13.4
SALISA [31]	Dense One-Stage	EfficientNet-B0	Tesla V100	—	—	1.39
SALISA [31]	Dense One-Stage	EfficientNet-B1	Tesla V100	—	—	3.2
FAM (D-FINE) [39]	DETR-based	D-FINE-S	A800	77.8	—	27.5
FAM (D-FINE) [39]	DETR-based	D-FINE-M	A800	84.9	—	59.9
FAM (D-FINE) [39]	DETR-based	D-FINE-L	A800	87.8	—	96.0
FAM (D-FINE) [39]	DETR-based	D-FINE-X	A800	91.0	—	207.4

— indicates a value not reported by the source study (NR), consistent with the data extraction protocol described in Section 3.6.

**Table 9:** Performance and efficiency metrics for studies evaluated on heterogeneous datasets, sorted alphabetically by model name. Speed and latency are reported in their original units (FPS and ms) to avoid compounding homogenization errors. Comparisons across studies are descriptive, given differing hardware platforms, datasets, and evaluation conditions. Similarly, speed values are not intended for direct cross-study ranking.

Model	Dataset	Arch.	Backbone	Hardware	mAP_50_	mAP_50–95_	Speed	FLOPs
BViTFPN [42]	Private	Dense	MobileNet	GTX 1080Ti	93.19	52.22	35.46 fps	—
CGFA-Net [30]	EventKITTI	Dense	ResNet-50	—	64.5	32.2	33.0 fps	—
EGSST-B [35]	Gen1	Graph	GNN + LViT	RTX 3090	—	44.6	4.6 ms	—
EGSST-E [35]	Gen1	Graph	GNN + LViT	RTX 3090	—	49.6	6.0 ms	—
EGSST-B-Y [35]	Gen1	Graph	GNN + LViT	RTX 3090	—	43.9	3.7 ms	—
EGSST-E-Y [35]	Gen1	Graph	GNN + LViT	RTX 3090	—	47.8	4.2 ms	—
EGSST-B [35]	1MPx	Graph	GNN + LViT	RTX 3090	—	45.4	5.1 ms	—
EGSST-E [35]	1MPx	Graph	GNN + LViT	RTX 3090	—	50.2	6.3 ms	—
EGSST-B-Y [35]	1MPx	Graph	GNN + LViT	RTX 3090	—	44.1	5.0 ms	—
EGSST-E-Y [35]	1MPx	Graph	GNN + LViT	RTX 3090	—	48.3	5.3 ms	—
SDFMD [41]	Private	DETR	—	—	—	—	—	—
SST [38]	Private	Dense	Swin	—	62.3	18.1	—	—
ST-Trans [36]	SIRSTD	Dense	C2FDark	RTX 4090	—	—	17.95 fps	—
TST-YOLOv8 [43]	CUCAFall	Dense	CSPDarknet	A100	—	—	155.7 fps/6.1 ms	24.8
TST-YOLOv8 [43]	DiverseFALL	Dense	CSPDarknet	A100	—	—	155.7 fps/6.1 ms	24.8

— indicates a value not reported by the source study (NR), consistent with the data extraction protocol described in Section 3.6. Architectural bins: Dense = Dense One-Stage, Graph = Graph-Based, DETR = DETR-Based. Backbone: GNN + LViT = GNN + LinearViT.

## Data Availability

The data supporting the findings of this review will be made publicly available via the review’s OSF registration (doi: 10.17605/OSF.IO/Q9PJ4) upon acceptance. Any additional data not included will be available from the corresponding author upon reasonable request. No custom code was used for the analysis. All materials used for the reproducibility of this study are available from the corresponding author upon reasonable request. This systematic review was not prospectively registered but was retrospectively registered in OSF/Generalized Systematic Review Registration Form under 10.17605/OSF.IO/Q9PJ4. The protocol was defined a priori following PRISMA 2020 guidelines, and the full registration details are available at https://osf.io/q9pj4/ (accessed on 2 September 2026).

## Supplementary Materials

The following supporting information can be downloaded at https://www.mdpi.com/article/10.3390/jimaging12090419/s1, File S1: PRISMA_2020_abstract_checklist; File S2: PRISMA_2020_checklist—for SLR submission; File S3: Database Search Strings.

## Appendix A. Methodology

This section contains the checklist related to study screening and selection. The checklist (Table A1) was applied to all candidate studies to ensure compliance with the research objectives. Verbose notes and comments were also kept for each entry but are omitted from the table for clarity.

**Table A1:** Screening checklist for study selection.

Criterion	Yes	No
Video object detection or sequential visual inference tasks (not still image detection only)?	□	□
Incorporates explicit temporal modeling, defined as any cross-frame information exchange mechanism during inference (e.g., feature aggregation, query propagation, recurrent state, or redundancy-based token selection across frames)?	□	□
Proposes quantifiable reductions in computational cost relative to stated baselines (e.g., reduced FLOPs, parameters, latency, or memory usage)?	□	□
Incorporates at least one complete transformer encoder or decoder block comprising multi-head self-attention, a position-wise feed-forward network, residual connections, and layer normalization, as defined in Vaswani et al. (2017) [26]?	□	□
Empirical study with experiments (not a review, survey, editorial, or perspective)?	□	□
Published in English?	□	□
Peer-reviewed journal or conference proceedings paper?	□	□
Reports relevant quantitative performance metrics (e.g., mAP, FPS, FLOPs, parameter count)?	□	□

## Appendix B. Results

**Table A2:** Studies excluded from the final synthesis due to lack of clear video modality or absence of temporal modeling (frame-by-frame inference only). Studies with no proposed model name are reported by first author name and publication year instead.

Study (Model)	Application	Dataset(s)
Fruit-YOLO [71]	Fruit Counting	Custom Video
FCTR [72]	Livestock Monitoring	ChickenFight-2024, MS COCO
Li et al. (2022) [73]	Fire/Smoke Detection	COCO, Mivia, Sy-fire
LAF-DETR [74]	Surveillance	SCB-Dataset3-S, CrowdHuman
DESPP-DETR [75]	Vehicle Detection	MS COCO 2017
ECVIST-YOLOv5 [76]	Mine Surveillance	Custom Video
FoT [77]	Sports Analysis	Soccer-Det, FIFA-Vid
HCLTYOLO [78]	Traffic Analysis	GTSDB, TT100K
FishViT [79]	Fish Detection	Custom Video
Zhang et al. (2023) [80]	UAVs	Ship Dataset (Kaggle)
Zhao et al. (2023) [81]	Fire Detection	Custom Video/image
LiVeDet [82]	Vessel Detection	Custom Vessel
MCFNet [83]	UAVs	Visdrone/DroneVehicle
MSO-DETR [84]	Maritime Search	SeaDronesSee, AFO
Trans-YOLOv8 [85]	VOD (General)	Custom dataset (Kaggle)
Lin-DETR-R50 [86]	Powerline Inspection	Custom Video
Zheng et al. (2024) [87]	Helmet Detection	Custom Video
SARViT [88]	Remote Sensing	BigEarthNet-MM, SpaceNet6
MFITN [89]	UAVs, IoVT	ISDD, CPLID, COCO
WeedSwin [90]	Agriculture	Alpha/Beta Weed Datasets

**Table A3:** Reviewer-derived quantitative values excluded from the primary synthesis (Table 8 and Table 9). These values are excluded from the primary synthesis and are not used in any comparative or ranking statement.

Model	Dataset	Architecture	Backbone	Hardware	mAP_50_	mAP_50–95_	Speed
ST-Trans [36]	SIRSTD	Dense	C2FDark	RTX 4090	64.9 ^*ι*^	—	17.95 fps
TST-YOLOv8 [43]	CUCAFall	Dense	CSPDarknet	A100	—	—	155.7 fps/6.1 ms
TST-YOLOv8 [43]	DiverseFALL	Dense	CSPDarknet	A100	—	—	155.7 fps/6.1 ms
SDFMD [41]	Private	DETR	—	—	93.0 ^⋆^	79.0 ^⋆^	35 fps ^⋆^

^*ι*^ indicates an mAP_50_ value reported under an IoU threshold not explicitly stated by the source study, inferred from dataset convention or model family defaults. ^⋆^ indicates a value not explicitly reported in text but approximated from a source study’s figures. TST-YOLOv8 uses a dynamic confidence threshold rather than a fixed IoU criterion; therefore, the reported mAP of 99.98 and 95.00 on CUCAFall and DiverseFALL cannot be converted to mAP_50_. Dense = Dense One-Stage, DETR = DETR-based.
