# Supplementary material for: Efficiency and Temporal Reasoning in Transformer-Based Video Object Detection: A Systematic Review
Source: J Imaging. 2026 Sep 5;12(9):419. doi: 10.3390/jimaging12090419 (PMC13608311; doi:10.3390/jimaging12090419)
Supplement: Supplementary file 1 [file jimaging-12-00419-s001.zip › Supplementary Material/Supplementary Material-Database Search Strings.pdf]

Search Strategy

Search Strings per Database

ACM

Search String with Database Syntax

```
 (
Title:(lightweight OR efficient OR "real?time" OR edge)
AND
Title:( video OR stream* OR sequence OR temporal )
AND
Title:( "object detection" OR "object recognition")
AND
Title:(transformer OR ViT)
)
OR
 (
Abstract:(lightweight OR efficient OR "real?time" OR edge)
AND
Abstract:( video OR stream* OR sequence OR temporal )
AND
Abstract:( "object detection" OR "object recognition" )
AND
Abstract:(transformer OR ViT)
)
```

Notes

- Prefilters:
    - Date:
        - 2020-2025
    - Content Type:
        - Research Article
- Databases Used:
    - ACM Guide to Computing Literature (3,933,427 records)
    - ACM Full-Text Collection (803,090 records)
- Search completed on:
    - 02/08/2025

IEEE Explore

Search String with Database Syntax

```
(( lightweight OR efficient OR "real?time" OR edge )
AND
( video OR stream* OR sequence OR temporal )
AND
```

```
( "object detection" OR "object recognition" )
AND
( transformer* OR ViT ))
```

Notes

- Filters Applied:
    - Year:
        - 2020-2025
    - Type:
        - Conferences
        - Journals
- Search completed on:
    - 06/08/2025

Science Direct

Search String with Database Syntax

```
( lightweight OR efficient OR "real?time")
AND
( video OR temporal )
AND
( "object detection" OR "object recognition" )
AND
( transformer OR ViT )
```

Notes

- Limitations:
    - Max Boolean Operators: 8
        - Weakest keywords cut to meet Boolean operator limit:
            - edge
            - stream*
            - sequence
- Advanced Search Input Field:
    - Title, abstract or author-specified keywords
- Prefilters:
    - Publication Year:
        - 2020-2025
- Search completed on:
    - 20/08/2025

Scopus

Search String with Database Syntax

```
TITLE-ABS-KEY ( ( lightweight OR efficient OR "real?time" OR edge )
AND ( video OR stream* OR sequence OR temporal )
```

```
AND ( "object detection" OR "object recognition" )
AND ( transformer* OR ViT ) )
AND PUBYEAR > 2019
AND PUBYEAR < 2026
AND ( LIMIT-TO ( DOCTYPE , "ar" )
OR LIMIT-TO ( DOCTYPE , "cp" ) )
AND ( LIMIT-TO ( LANGUAGE , "English" ) )
```

Notes

- Field Searched (Through Advanced Search):
  - Article title, Abstract, keywords
- Prefilters:
  - Date:
    - 2020-2025
  - Document Type:
    - Article
    - Conference
  - Language:
    - English
- Search completed on:
  - 14/08/2025

Springer Nature

Search String with Database Syntax

```
(title-abs-key:(lightweight OR "real?time" OR edge))
AND  title-abs-key:( video OR stream* OR sequence OR temporal ) )
AND  (title-abs-key:("object detection" OR "object recognition"))
AND  (title-abs-key:(transformer OR ViT))
```

Notes

- Prefilters:
  - Content Type:
    - Article
    - Research Article
  - Language:
    - English
  - Date:
    - 2020-2025
- Search completed on: 25/08/2025

GENERAL NOTES

Some databases provide the option to export the string with the applied filters and specific queried fields embedded in them. Where applicable, this feature was used to provide the exact internal syntax of the used database.
